# Value of loop electrosurgical excision procedure conization and imaging for the diagnosis of papillary squamous cell carcinoma of the cervix

**DOI:** 10.3389/fonc.2023.1166818

**Published:** 2023-07-05

**Authors:** Can Cui, Ziren Chen, Lingxiao Luo, Jianping Zeng, Xiaoyi Sun, Long Sui, Congjian Xu, Zhongpeng Fu, Qing Cong

**Affiliations:** ^1^ Obstetrics and Gynecology Hospital of Fudan University, Shanghai, China; ^2^ Shanghai Key Laboratory of Female Reproductive Endocrine Related Diseases, Fudan University, Shanghai, China

**Keywords:** cervical cancer, imaging, loop electrosurgical excision procedure conization, papillary squamous cell carcinoma, hysterectomy and postoperative pathology

## Abstract

**Background:**

Loop electrosurgical excision procedure (LEEP) conization and hysterectomy are performed for some patients with papillary squamous cell carcinoma (PSCC), whereas only hysterectomy is performed for others. We aimed to determine the optimal management for PSCC.

**Methods:**

Patients diagnosed with PSCC by colposcopy-directed biopsy between June 2008 and January 2020 who underwent LEEP conization and hysterectomy or only hysterectomy at our hospital were enrolled. Results of cervical cytology, high-risk human papillomavirus testing, transvaginal sonography, pelvic magnetic resonance imaging, LEEP, hysterectomy, and pathology testing of colposcopy-directed biopsy samples were analyzed.

**Results:**

A total of 379 women were diagnosed with PSCC by colposcopy-directed biopsy; 174 underwent LEEP before hysterectomy and 205 underwent only hysterectomy. Patients underwent and did not undergo LEEP were aged 47 ± 11 years and 52 ± 11 years, respectively. Among women who underwent LEEP, the agreement between LEEP and hysterectomy pathology was 85.1%. For women who underwent only hysterectomy, the agreement between preoperative clinical staging and pathological staging after hysterectomy was 82.4%. For patients with preoperative imaging indicative of malignancy, the accuracy of LEEP for diagnosing and staging PSCC was 88.5%, whereas for the hysterectomy-only group, it was 86.2%. For patients without malignancy detected with imaging, the accuracy of LEEP for diagnosing and staging PSCC was 81.6%; however, for those who did not undergo LEEP, it was 70.0%.

**Conclusion:**

For women diagnosed with PSCC by colposcopy-directed biopsy, LEEP conization is necessary for an accurate diagnosis when imaging does not indicate cancer; however, LEEP is not necessary when imaging indicates cancer.

## Introduction

Cervical cancer is the second most common malignant gynecological cancer worldwide after breast cancer. In 2018, a total of 569,847 new cases of cervical cancer were reported (1). There are three main pathological types of cervical cancer: squamous cell carcinoma, which is the most common; adenocarcinoma; and adenosquamous carcinoma. Papillary squamous cell carcinoma (PSCC) of the cervix is a rare and distinct form of cervical carcinoma (2) that accounts for 1.6% of all cervical carcinomas (3). PSCC grows superficially, with wart-like or exophytic features (2). Detection of stromal invasion using biopsy is difficult because of the papillary growth of the tumor (4). In clinical practice, some women with PSCC are advised to undergo hysterectomy directly after biopsy, whereas others are advised to undergo cervical conization before hysterectomy.

Cervical cytology and high-risk human papillomavirus (HPV) screening are the most important methods used for the early detection of cervical cancer and precancerous lesions. During the past 50 years, colposcopy has had a key role in reducing the incidence of and mortality caused by cervical cancer. Loop electrosurgical excision procedure (LEEP) conization can further detect early invasive cancers that cannot be detected by visual inspection or colposcopy-directed biopsy because it is a more comprehensive and accurate histological examination (5, 6). Additionally, imaging can help clinicians detect the invasiveness of the lesion and estimate its size. When performing local staging before treatment, imaging *via* transvaginal ultrasound (TVS) and/or magnetic resonance imaging (MRI) are critical for defining the pelvic extent of the tumor because they can allow more accurate assessments of the tumor size, stromal invasion depth, and parametrial invasion (7).

Due to the exophytic growth of PSCCs it is difficult to detect stromal invasion of tumors using biopsy. However, it is important to know whether there is a stromal invasion before hysterectomy, and the difference in the depth of invasion will affect the clinical stage of the patient. For patients with different stages, the surgical method also varies greatly. Therefore, LEEP is needed to help further determine the presence and depth of infiltration. While in clinical practice, doctors may decide to omit LEEP and perform hysterectomy directly due to various factors such as cervical atrophy, obvious macroscopic mass at the cervix or imaging shows tumor invasion. We aimed to determine whether LEEP is necessary when imaging indicates PSCC-type malignancy. The accuracy rates of LEEP conization and imaging for PSCC staging are unknown. Therefore, we also aimed to explore the accuracy rates of LEEP and imaging for the staging and optimal management of PSCC.

## Materials and methods

### Patient selection

The inclusion criterion was a diagnosis of PSCC by colposcopy-directed biopsy results. The exclusion criteria were: 1) clearly significant stromal invasion demonstrated by colposcopy-guided biopsy; 2) loss to follow-up after LEEP conization or with incomplete pathological reports. The data of cervical cytology/high-risk HPV screening results, colposcopy-directed biopsy results, LEEP outcomes, imaging results, hysterectomy outcomes, and postoperative pathology results were retrieved and evaluated.

### Patients

Patients who were diagnosed with PSCC by colposcopy-directed biopsy at OGHFU from June 1, 2008, to January 31, 2020, were enrolled. At OGHFU, patients with abnormal cervical cytology or positive high-risk HPV test results are referred for a colposcopy within 2–6 weeks. Colposcopy-directed biopsy was performed by experienced colposcopists. During this period, a total of 38,220 patients underwent LEEP for cervical precancer or a worse diagnosis. A total of 2,268 patients underwent hysterectomy for stage IA1 cervical cancer and 9,444 underwent modified or radical hysterectomy for stages IA2 to IIA1 cervical cancer.

### Cytology and high-risk HPV testing

We used liquid-based cytology (SurePath; Becton, Dickinson and Company, Franklin Lakes, NJ, USA) for cytology testing. For high-risk HPV testing, we used the Cobas 4800 assay (Roche, Penzberg, Germany) or the fluorescence-based multiplex real-time HPV DNA genotyping kit (Bioperfectus, Jiangsu, China), which can detect high-risk HPV types 16, 18, 31, 33, 35, 39, 45, 51, 52, 56, 58, 59, 66, and 68.

### LEEP conization and pathological examination

All procedures were performed by 1 of the 18 experienced colposcopists at OGHFU. Different diathermy loops were used for excision based on the size of the cervical lesion and the location of the transformation zone. All excisions were performed under colposcopic guidance. The cervical transformation zone and lesion were excised to an adequate scale, extending 4–5 mm beyond the lesion in most cases. The tissues were excised to depths of 7–10 mm, 10–15 mm, and 15–25 mm for type I, type II, and type III cervical transformation zones, respectively. A second pass with a small loop was also performed to obtain an endocervical specimen for further histological evaluation. The loop size and volume, length, and thickness of the cone specimen were recorded. The pathologists cut each cone tissue sample into 12 pieces and embedded each piece onto a paraffin block. Both ectocervical and endocervical margins were clearly examined and reported by the pathologists. All pathological specimens were processed using a standardized protocol, interpreted by experienced colposcopists, and verified by another experienced pathologist (6).

### Imaging

All patients who were diagnosed with PSCC underwent preoperative imaging before surgery. We used TVS and/or MRI to assess the depth of tumor invasion and metastasis. All imaging examinations were completed by an attending physician or a senior physician with imaging experience; the results were reviewed by another physician with imaging experience.

### Hysterectomy and postoperative pathology

Surgery was performed by two experienced gynecologists. The tissue excised during surgery was sent to the pathology department for examination by two experienced pathologists.

### Statistical analyses

Approval was obtained from the Institutional Review Board of OGHFU before data extraction was performed. Statistical analysis was performed using the Pearson chi-square test, t test, and logistics regression analysis using Stata 15.1 (Stata Corp, LLC, Texas, USA); a p-value of <0.05 was considered statistically significant.

## Results

Between June 2008 and January 2020, a total of 607 women were diagnosed with PSCC by colposcopy-directed biopsy results. However, 159 women diagnosed with invasive cancer based on multiple biopsies were excluded, and 69 women were excluded because of loss to follow-up or incomplete pathological results. Finally, 379 women diagnosed with PSCC using colposcopy-directed biopsy were included in the study. Among them, 174 women underwent LEEP before hysterectomy and 205 underwent hysterectomy without LEEP.

The characteristics of women with PSCC are shown in **Table 1**. Women in the LEEP plus hysterectomy group underwent LEEP followed by hysterectomy. Women in the hysterectomy group underwent hysterectomy without LEEP. The mean age of the women was 47.1 ± 10.9 years and 51.8 ± 11.0 years) in the LEEP plus hysterectomy group and hysterectomy group, respectively; the difference was statistically significant (p<0.001). Additionally, 86.8% in the LEEP plus hysterectomy group and 87.0% in the hysterectomy groups had high-risk HPV. Among the patients in the LEEP plus hysterectomy group, according to the liquid-based cytology report, 28 had low-grade squamous intraepithelial lesions (LSIL) and 133 had high-grade squamous intraepithelial lesions (HSIL); in the hysterectomy group, 31 had LSIL and 164 had HSIL, and the relevant information was unavailable for the others. Furthermore, in the LEEP plus hysterectomy group, 61 were diagnosed with cancer based on imaging results and 76 had negative imaging results for cancer. In the hysterectomy group, 130 were diagnosed with cancer based on imaging results and 50 had negative imaging results for cancer; these differences were statistically significant (p<0.001). Finally, cervical HSIL, stage IA1, stage IA2, and stage ≥IB1 were diagnosed in 19.3% (73/379), 10.6% (40/379), 3.4% (13/379), and 66.8% (253/379) of the patients with PSCC, respectively. In all patients with PSCC, the maximum diameter of cervical lesions was compared between the two groups. In the LEEP plus hysterectomy group, the maximum diameter of cervical lesions was 1.03 ± 1.56 cm. In the hysterectomy group, the maximum diameter of the lesions was 1.94 ± 1.79 cm; the difference was statistically significant (p<0.0001) (**Table 1**). There was no obvious cervical lesion in patients with negative imaging results for malignant lesions (diameter=0 cm).

**Table 1:** Clinical characteristics of women with cervical papillary squamous cell carcinoma.

Clinical characteristics	Total	LEEP+hysterectomy (n=174)	Hysterectomy (n=205)	p-value
Age, years (mean ± SD)	49.4 ± 11.2	47.1 ± 10.9	51.8 ± 11.0	<0.001
hrHPV	86.9%	86.8%	87.0%	>0.999
Cytology				
≤LSIL	59 (16.6%)	28 (17.4%)	31 (15.9%)	0.706
≥HSIL	297 (83.4%)	133 (82.6%)	164 (84.1%)	
Imaging				
Suspicion of cancer	191 (60.3%)	61 (44.5%)	130 (72.2%)	<0.0001
Negative	126 (39.7%)	76 (55.5%)	50 (27.8%)	
Maximum diameter of cervical lesion	1.51 ± 1.74	1.03 ± 1.56	1.94 ± 1.79	<0.0001
Staging				
≤HSIL	73 (19.3%)	46 (26.4%)	27 (13.2%)	<0.0001
IA1	40 (10.6%)	30 (17.2%)	10 (4.9%)	
IA2	13 (3.4%)	9 (5.2%)	4 (2.0%)	
≥IB1	253 (66.8%)	89 (51.1%)	164 (80.0%)	

Available hrHPV, cytology, and imaging results are described. LEEP, loop electrosurgical excision procedure; hrHPV, high-risk human papillomavirus; SD, standard deviation; HSIL, high-grade squamous intraepithelial lesion or worse; LSIL, low-grade squamous intraepithelial lesion.

**Figure 1:**
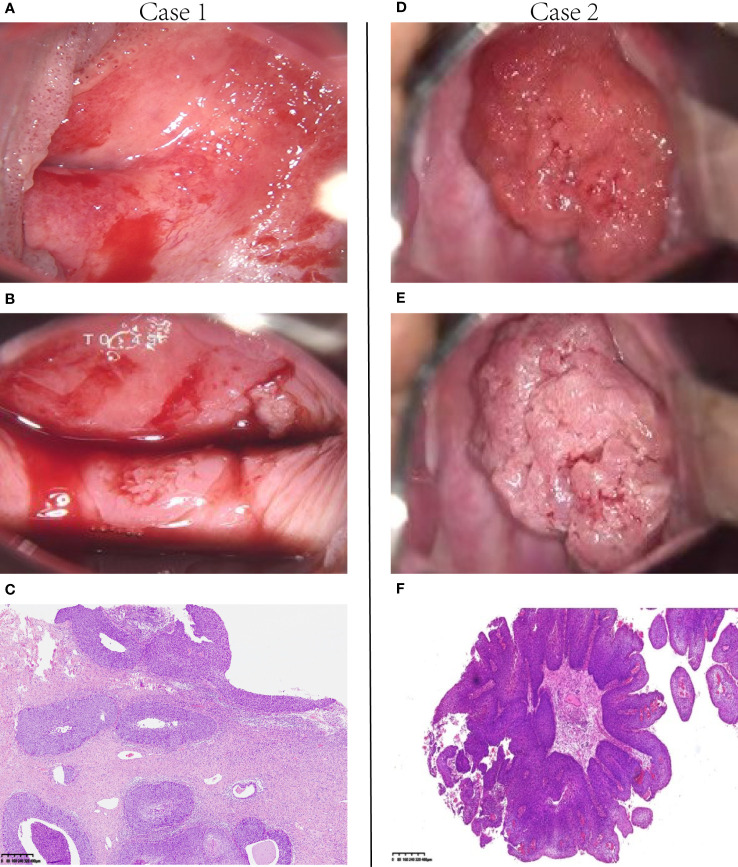
Two representative cases. **(A, B)** Case 1. Colposcopic photographs show that the lesions extended from the cervix to the vagina. **(C)** The surgical specimen from this patient showed noninvasion; the lesion extended to the basement membrane, which was predominant over the papilla-like exophytic growth (H&E, 40×). Therefore, it was diagnosed as papillary squamous cell carcinoma *in situ*. **(D, E)** Case 2. A typical papilla-like exophytic lesion was observed under the colposcope. **(F)** In this patient, the tumor tissue appeared PSCC-like according to the selective colposcopic biopsy results (H & E, 4×). H&E, hematoxylin and eosin; PSCC, papillary squamous cell carcinoma.

We present two cases, both of which were diagnosed with exophytic papillary squamous cell carcinoma prior to hysterectomy, where stromal invasion could not be determined due to superficial biopsy tissue. Case 1 was diagnosed with HSIL after radical hysterectomy. For case 2, the postoperative pathology results showed invasive squamous cell carcinoma (**Figure 1**). Clinicopathological features were assessed using univariate logistic regression of PSCC with invasive and noninvasive cervical lesions (**Table 2**). Imaging indicated that a malignant tumor (p<0.0001) and the maximum diameter of the cervical lesion (p=0.012) were statistically significant for PSCC with an invasive cervical lesion according to univariate logistic regression. Other variables, including age (p=0.39), high-risk HPV infection (p=0.99), and Leydig cell tumors, indicated that a malignant tumor (p=0.34) was not statistically significant for PSCC with an invasive cervical lesion. Candidate predictors, including age, imaging-indicated malignant tumors, and the maximum diameter of cervical lesions, were analyzed by multivariate logistic regression. The results showed that the maximum diameter of cervical lesions (odds ratio: 2.561; 95% confidence interval: 1.246-5.263; p=0.011) was statistically significant, that is, it is an independent risk factor for PSCC with invasive cervical lesion. The Hosmer-Lemeshow goodness-of-fit test yielded a non-significant statistic (p=0.227).

**Table 2:** Multivariate logistic regression analysis of different clinicopathological features and invasive status of PSCC patients.

Factors	Invasive cervical cancer	
	OR (95% CI)	p-value
Age	1.022 (0.993-1.052)	0.138
Imaging-indicated malignant tumor	1.495 (0.292-7.655)	0.63
Maximum diameter of cervical lesion	2.561 (1.246-5.263)	0.011

OR, odds ratio; CI, confidence interval.

**Table 3** shows the histopathological staging of cancer in women with PSCC who underwent LEEP before hysterectomy. The histopathological results after hysterectomy matched those detected by LEEP in 85.1% of cases. The results of LEEP histopathology indicating HSIL and histopathological results after hysterectomy matched in 70.8% of the cases, those for stage IA1 matched in 79.3% of cases, and those for stage IA2 matched in 85.7% of cases. The accuracy of LEEP histopathology indicating stage ≥IB1 was 100% when compared with the histopathological results after hysterectomy.

**Table 3:** Histopathological staging of cervical papillary squamous cell carcinoma with LEEP before hysterectomy.

LEEP histopathology	Total (%)	Post-hysterectomy histopathology				Final histopathology agreed with LEEP	Accuracy
		≤HSIL	IA1	IA2	≥IB1		
≤HSIL	65 (37.4%)	46	7	2	10	46	70.8%
IA1	29 (16.7%)	16	7	1	5	23	79.3%
IA2	7 (4.0%)	4	1	1	1	6	85.7%
≥IB1	73 (42.0%)	25	0	1	47	73	100.0%
Total (%)	174 (100.0%)	91 (52.3%)	15 (8.6%)	5 (2.9%)	63 (36.2%)	148	85.1%

Among women who underwent hysterectomy without LEEP, 178 underwent a radical hysterectomy, 1 underwent a modified radical hysterectomy, and 26 underwent a total hysterectomy (**Table 4**). According to the histopathological results, the accuracy rates of the selection of surgical methods for radical hysterectomy, modified hysterectomy, and total hysterectomy (according to National Comprehensive Cancer Network cervical cancer guideline 2020) were 86.5% (154/178), 0% (0/1), and 57.7% (15/26), respectively; the total accuracy rate was 82.4% (169/205). **Table 5** shows the histopathology results after LEEP and hysterectomy stratified by surgical methods for all patients with PSCC and positive imaging results for cancer. For women whose imaging results were suspicious for cancer, the accuracy rate of LEEP based on the histopathological results after LEEP and hysterectomy was 88.5% (54/61); however, the accuracy rate of hysterectomy without LEEP was 86.2% (112/130). These results were similar to the total accuracy rates of LEEP (88.5%) (p=0.2673). **Table 6** shows the LEEP and hysterectomy histopathology results of all patients with PSCC with imaging results negative for carcinoma. The accuracy rate of LEEP before hysterectomy was 81.6% (62/76), which was significantly higher than the accuracy rate of hysterectomy without LEEP (70.0%; 35/50).

**Table 4:** Post-hysterectomy histopathological staging of cervical papillary squamous cell carcinoma for patients who did not undergo LEEP.

Surgical method	Total (%)	≤HSIL	IA1	IA2	≥IB1	Accuracy
Radical hysterectomy	178 (86.8%)	17	4	3	154	86.5%
Total hysterectomy	26 (12.7%)	9	6	1	10	57.7%
Modified radical hysterectomy	1 (0.5%)	1	0	0	0	0.0%
Total (%)	205 (100.0%)	27 (13.2%)	10 (4.9%)	4 (2.0%)	164 (80%)	82.4%

**Table 5:** Accuracy of LEEP for diagnosing PSCC and staging for women whose imaging results indicated a malignant tumor before surgery.

Imaging			Total	Post-hysterectomy Histopathology				Accuracy
				≤HSIL	IA1	IA2	≥IB1	
Suspicion of Ca	LEEP (n=61)	≤HSIL	9	4	2	0	3	88.5% (54/61)
		IA1	3	2	0	0	1	
		IA2	3	0	1	1	1	
		≥IB1	46	11	0	1	34	
	Surgery (n=130)	Radical hysterectomy	120	9	1	1	109	86.2% (112/130)
		Modified radical hysterectomy	1	0	0	0	1	
		Total hysterectomy	9	2	1	0	6	

**Table 6:** Accuracy of LEEP for diagnosing PSCC and staging for women whose preoperative imaging results were negative for cancer.

Imaging			Total	Post-hysterectomy Histopathology				Accuracy
				≤HSIL	IA1	IA2	≥IB1	
Negative	LEEP (n=76)	≤HSIL	39	30	4	2	3	81.6% (62/76)
		IA1	18	12	1	1	4	
		IA2	4	4	0	0	0	
		≥IB1	15	10	0	0	5	
	Surgery (n=50)	Radical hysterectomy	35	7	3	1	24	70.0% (35/50)
		Modified radical hysterectomy	0	0	0	0	0	
		Hysterectomy	15	6	5	1	3	

## Discussion

PSCC grows superficially in an exophytic manner (8),this pattern of growth is different from that of traditional squamous cervical cancer (9). Few studies of PSCC have been conducted, and their sample sizes ranged from 1 to 55 cases (3, 4, 10–13). Moreover, the final staging of PSCC is unknown. To the best of our knowledge, this is the largest retrospective study of PSCC (379 cases). In our study, the final diagnoses were confirmed after LEEP plus hysterectomy or hysterectomy without LEEP; 19.3% of the cases involved HSIL and 80.7% of the cases involved stage IA1 (10.6%), stage IA2 (3.4%), and stage ≥IB1 (66.8%) cervical cancer. This study found that when the imaging results were negative for cancer, the accuracy of LEEP was higher and more critical than that of hysterectomy without LEEP (81.6% vs. 70%; p=0.13). Although the p-value of 0.13 indicates no statistically significant difference, it has considerable clinical significance. LEEP improves the diagnostic accuracy by more than 10%. However, when imaging indicated cancer, the accuracy of hysterectomy without LEEP and that of hysterectomy after LEEP were nearly identical (88.5% vs. 86.2%; p=0.267). Therefore, LEEP is not necessary when imaging indicates cancer in women with PSCC.

**Figure 2:**
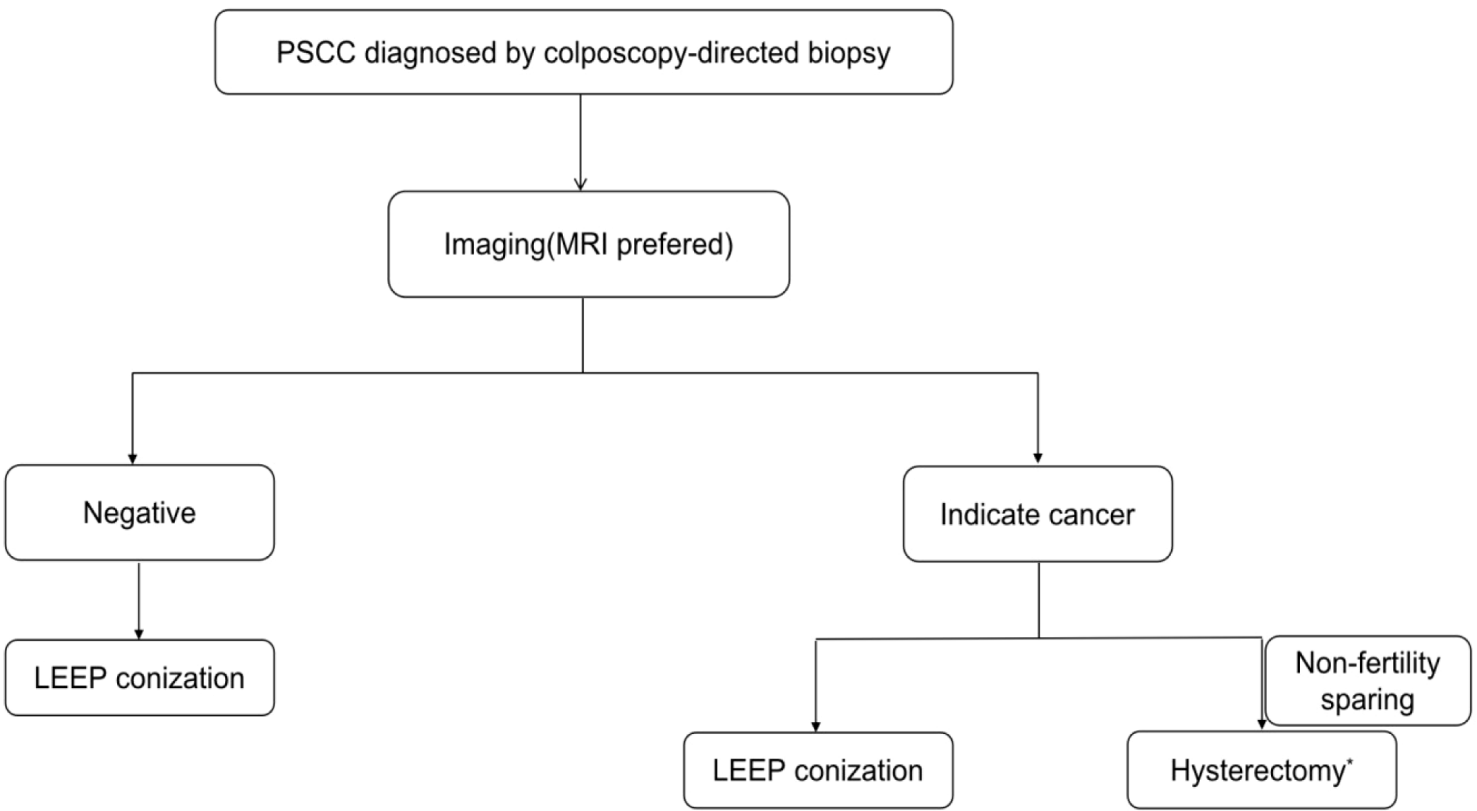
Recommended algorithm for the management of PSCC. *Hysterectomy includes total hysterectomy, radical hysterectomy, and modified radical hysterectomy.

When imaging indicated cancer, three of nine women with LEEP pathology had HSIL and one of three women with LEEP pathology indicating stage IA1 were finally diagnosed with stage IB1 after hysterectomy. A total of 6.6% (4/61) of patients underwent inadequate surgical resection, indicating that the papillary exophytic pattern made it difficult to reach the deeper component of PSCC with LEEP. Radical hysterectomy without LEEP was excessive for 10.8% (14/130) of patients with imaging results indicating cancer. Based on our study, we suggest an algorithm for the management of PSCC (**Figure 2**). For PSCC diagnosed with colposcopy-directed biopsy, pelvic MRI is recommended (14). If imaging results indicate cancer, then individualized treatment is extremely important. Before surgery, the patients must be completely informed about the choices and risks of surgical options. Even if LEEP is performed before surgery, it is possible that a second surgery or supplementary radiotherapy and chemotherapy might be required, especially if the lesions are diagnosed as IA1 stage and precancerous by LEEP. However, direct extensive total hysterectomy without LEEP might be an overtreatment for 10% of the cases; hence, radical hysterectomy can be performed as a nonfertility-sparing approach with informed consent (15, 16). If imaging results are negative, then cervical conization should be performed for precise treatment (17). Although enhanced MRI cannot 100% demonstrate the depth of invasion, our research shows the importance of imaging examination for guiding the treatment of PSCC. Nagura et al. also proposed the important role of MRI for the precise diagnosis of PSCC (2). Other studies have suggested that patients with visible lesions should directly undergo radical hysterectomy; however, because of the small sample size, the level of evidence might be low (7, 13, 18, 19).

PSCC shows more favorable prognosis than other cervical invasive cancers, but visible masses are common in PSCC. According to NCCN guidelines and existing literature, one of the indications for fertility-sparing approach is that the maximum diameter of the tumor is ≤ 2cm, and for 2-4cm, the fertility-sparing approach can be performed selectively. As our results showed that maximum diameter of cervical lesions is an independent risk factor for PSCC with invasive cervical lesion. The indications for fertility-sparing in the guidelines are also appropriate for PSCC. The study has shown that surgical treatment of cervical was associated with poor obstetric outcomes (20). However, given the specificity of PSCC, the effect on fertility after cervical surgery in patients with PSCC requires more data to analyze. Especially for PSCC patients have no stromal infiltration despite the presence of relatively large visible masses, whether changes in the internal environment after pregnancy will have an impact on PSCC requires more research.

There are some limitations to this study. LEEP conization was performed for patients with PSCC; however, cold-knife conization might be a better choice. All patients with PSCC were examined using TVS, but only half (52.0%; 197/379) examined using pelvic MRI with contrast. Because imaging is important for the preoperative evaluation of PSCC, pelvic MRI is preferred. Another limitation was the retrospective design of the study. Therefore, we could not obtain complete information about the medical history of the patients. Additionally, the retrospective nature might have introduced selection bias and misclassification or information bias. In the future, large prospective studies are necessary to generate stronger evidence.

## Conclusions

To the best of our knowledge, this is the largest retrospective study of PSCC. Our research summarized the clinical and pathological characteristics of PSCC and showed the importance of imaging examination for guiding the treatment of PSCC with large sample size. Our results also showed that when women diagnosed with papillary squamous cell carcinoma *via* colposcopy-directed biopsy, loop electrosurgical excision procedure (LEEP) conization is necessary for accurate diagnosis when imaging is negative for cancer; however, LEEP is not necessary when imaging indicates cancer.

## Data availability statement

The raw data supporting the conclusions of this article will be made available by the authors, without undue reservation.

## Ethics statement

The study was conducted in accordance with the Declaration of Helsinki and approved by the ethics committee of the Obstetrics and Gynecology Hospital of Fudan University (IRB Number: 2017-11). Written informed consent was obtained from the individual(s) for the publication of any potentially identifiable images or data included in this article.

## Author contributions

QC, CC, and ZF provided the conception. CC and ZC collected, analyzed and interpreted the patient data. CC was a major contributor in writing the manuscript. All authors read and approved the final manuscript. QC made the main contribution to the funding acquisition. CX, ZF, and QC contributed to supervision and investigation. All authors contributed to the article.
